# Brovalol, a Distinct Advance in Valerian Therapy

**Published:** 1910-12

**Authors:** 


### Brovalol, a Distinct Advance in Valerian Therapy

According to clinical experiments, made recently in Professor v. Bardeleden's Gynecological Polyclinic in Berlin, by Dr.

Theodore Kuttner, Assistant Physician, brovalol has proved an extremely valuable therapeutic agent in disorders of the central and sympathetic nervous system. The results obtained by him confirm in every way those previously reported by Prof. Grawitz's Division of the Charlottenburg-Westend Hospital, Berlin. (Dr. Maeder, *Therapeutische Monatshefte,* October, 1908).

The best results with brovalol were obtained in neurasthenia, hysteria, excitability of the nervous system, in the psycho-neuroses incidental to onanism and Basedow's disease, in climacteric disorders and in the menopause after hysterectomy with castration.

In gastric neuroses with vomiting from local or reflex causes, as for instance during pregnancy and in seasickness, two to three brovalol pearls, taken several times a day, followed by a slowly sipped cup of black coffee, have rendered excellent service.

The author cites four typical cases, one of which a case of morphinism, the good results of which give rise to the possibility that brovalol will also prove a valuable adjuvant in the treatment of drug addictions.

It is stated with particular satisfaction that brovalol was always readily taken and well borne by the patients, the absence of the objectionable eructations, common to other valerian preparations, being emphasized as one of its chief virtues.

The successful chemical combination of bromine with isovaleric-acid-borneolester, achieved in the production of brovalol, has not only increased the nervine and sedative properties of the most important constituent of valerian, but places at the disposal of the physician an unusually highly concentrated valerian preparation. The author demonstrates this latter fact by means of comparative figures which show that five to six brovalol pearls correspond in their contents of isovaleric-acid-borneolester to two and one-fifth pounds of valerian root.

